# Editorial: Rising stars in brain imaging and stimulation 2023

**DOI:** 10.3389/fnhum.2024.1437975

**Published:** 2024-06-07

**Authors:** Samar S. Ayache, Mana Biabani, Moussa A. Chalah

**Affiliations:** ^1^Department of Neurology, Gilbert and Rose-Marie Chagoury School of Medicine, Byblos, Lebanon; ^2^Institut de la Colonne Vertébrale et des NeuroSciences (ICVNS), Centre Médico-Chirurgical Bizet, Paris, France; ^3^EA4391 Excitabilité Nerveuse and Thérapeutique, Université Paris Est Créteil, Créteil, France; ^4^Department of Clinical Neurophysiology, DMU FIxIT, Henri Mondor University Hospital, Assistance Publique-Hôpitaux de Paris (APHP), Créteil, France; ^5^Turner Institute for Brain and Mental Health, School of Psychological Sciences, Monash University, Clayton, VIC, Australia; ^6^Pôle Hospitalo-Universitaire Psychiatrie Paris 15, GHU Paris Psychiatrie et Neurosciences, Hôpital Sainte-Anne, Paris, France

**Keywords:** neuroimaging, neuromodulation, brain stimulation, transcranial magnetic stimulation, transcranial electric stimulation, NIBS

## 1 Introduction

Our current knowledge of the brain and behavior largely owes its depth to the remarkable advances in neuromodulation and neuroimaging in recent decades. The era of modern brain stimulation likely began with the pioneering work of Giovanni Aldini in 1804, which demonstrated contraction of facial muscles in response to the electrical stimulation of the exposed cortex (Parent, 2004). Since then, there has been growing interest in the use of electricity in the human brain for investigative and therapeutic purposes, which rapidly led to the development of safer and more adaptable methods such as non-invasive brain stimulation (Lozano and Mark, 2013). Such techniques allow for controlled manipulation of neural circuits *in vivo*, offering a unique tool to investigate causal mechanisms in the human brain and trigger therapeutic changes in pathological conditions (Bergmann and Hartwigsen, 2021). Neuroimaging, another crucial tool in neuroscience, refers to an umbrella term for various methods that visualize and map the function or structure of the nervous system. Brain imaging is currently in wide use to elucidate the neural correlates of human behavior by revealing *where* and *when* activity occurs in the brain (Otte and Halsband, 2006).

Integrating brain stimulation with imaging enables the investigation of causal mechanisms underlying brain dynamics with exceptional spatiotemporal resolution, as well as optimizing therapeutic interventions. However, despite remarkable methodological advances, the field is challenged by some major limitations. For instance, the mechanisms of action of many stimulation techniques are not fully understood (Bergmann et al., 2016). Also, there is a large heterogeneity in responses to brain stimulation across individuals, which necessitates personalized approaches to optimize exploratory and therapeutic outcome (Cash and Zalesky, 2024). Furthermore, combining neuroimaging with brain stimulation is technically challenging due to the risk of various types of artifacts which can obscure imaging measures and complicate the results interpretation (Peters et al., 2013; Rogasch et al., 2022). This Research Topic presents a selection of novel research addressing the existing challenges in the field with the aim of enhancing the precision and efficacy of interventions and assessments.

## 2 Neuroimaging and neuromodulation

### 2.1 Exploring disease pathophysiology or evaluating physiological changes following interventions

To start, Ritter et al. assessed the somatosensory representation of deafferented limbs in unilateral transtibial amputees and matched healthy controls. In patients, transcutaneous electrical stimulation of the truncated peroneal nerve resulted in the activation of functionally preserved cortical representation of the amputated limbs documented using magnetoencephalography (MEG). The MEG-derived dipole was localized in the somatosensory cortex with no significant difference in the dipole characteristics between patients and healthy controls, or between both sides in patients. These results suggest the potential role of using the truncated nerve in the field of rehabilitation to restore sensory feedback or motor control over a prosthesis. In another work, Ayache and Chalah reappraised the application of noninvasive brain stimulation and neuroimaging tools to explore the mechanisms of silent symptoms in multiple sclerosis (MS). This includes MS fatigue, affective symptoms, cognitive deficits, pain, and sleep disorders. The authors have summarized the literature linking these abnormalities with specific dysfunctions in brain circuits and neurotransmission.

Besides the exploration of the underlying pathways in diseases, neuroimaging and neuromodulation can be used to explore the neuroscientific underpinning of some behaviors or interventions. For instance, Sakurai and colleagues appraised the neural correlates of autonomous sensory meridian response (ASMR) which entails a sensory response resulting from exposure to audiovisual stimuli. Healthy adults underwent functional MRI while watching ASMR and control videos. Compared to control and unliked ASMR videos, only liked ASMR videos resulted in a significant activation of the amygdala, the insula, and the frontal cortex which are involved in the autonomic and limbic systems. ASMR videos might result in functional brain changes in emotion-related areas and their use might have a utility in the mental health field. In another work, Perrey discussed the available techniques that could be used to unveil the functional cerebral underpinnings of resistance training. While electroencephalography (EEG) and functional near-infrared spectroscopy could help in understanding the neural correlation of a change (e.g., brain waves, oxygenation levels), transcranial current stimulation could enable modulating the brain function and assessing the behavioral outcomes of such an intervention.

It is noteworthy that for the exploratory use of transcranial magnetic stimulation (TMS), it is crucial to optimize the measured outcomes. TMS coupled with EEG constitutes an interesting exploratory tool. However, it is important to remove TMS artifacts to enhance the quality and interpretation of the obtained data, especially regarding early latency. In this context, Mutanen et al. ran a study comparing the performance of two data-processing strategies: combined signal-space projection–source-informed reconstruction approach and independent component analysis. While the former might have more advantage when dealing with artifacts that are time-locked to TMS pulse, the latter still constitutes a pertinent tool, especially with artifacts that are not time-locked to TMS stimulus. In the same logic, optimizing the TMS cerebral targeting method would allow better study outcomes. Here, Uehara et al. evaluated the difference between mean Talairach coordinates issued from healthy adults and the actual location of the hand motor area in adult patients using neuronavigated TMS. Motor cortex stimulation using the Talairach coordinate system did not induce EMG-motor evoked potentials in half of the sample, whereas stimulating the anatomical hand motor area was able to yield such effects. The mean and the maximal scalp distance between both locations were 6.1 mm and 19.5 mm, respectively. Therefore, using generalized coordinates might not provide optimal outcomes in TMS trials. Research employing other techniques might be of help.

### 2.2 Improving functions and alleviating symptoms

Ayache and Chalah suggested the potential benefits of using noninvasive brain stimulation techniques [electroconvulsive therapy (ECT), repetitive TMS, and transcranial electric stimulation (tES)] to alleviate MS symptoms. While ECT appears to have good safety and efficacy in treating psychiatric symptoms in MS, tES and TMS might also have their place in treating some MS symptoms (e.g., fatigue). Also, Schellen et al. applied cerebellar transcranial alternating current stimulation coupled with fear extinction training in a randomized sham-controlled parallel trial involving healthy participants. Neither stimulation arm had effects on fear extinction or recall. The negative results warrant further studies on the optimal stimulation parameters or protocol design. Moreover, Perray highlighted the need for further research assessing the utility of transcranial current stimulation as a tool to improve exercise capacity in the field of sport training and injuries prevention.

## 3 Discussion

This topic shed light on the importance of neuroimaging and neuromodulation techniques as tools to increase scientific insights into brain mechanisms, brain diseases, and responses to interventions. As seen in some of the studies, there is an attempt to improve the collected outcomes or the treatment response. Here, several points merit to be addressed. The considered techniques could enable identifying predictors of treatment response or resistance (Dunlop et al., 2019; Runia et al., 2022). In addition, the use of functional MRI might have its place in the development of an individualized neuronavigated neuromodulation as suggested by the promising experience obtained in depression (Fox et al., 2012; Caulfield et al., 2022). Moreover, taking into consideration the dose-dependent pattern of response to neuromodulation seen in some conditions (Hutton et al., 2023), a higher number of stimulation sessions or longer protocols might have their utility in optimizing the clinical outcomes. Furthermore, combining neuroimaging and neuromodulation techniques could help unravel the brain functions and responses to interventions. Finally, a multidisciplinary approach integrating different modalities might contribute to enhancing the management of patients with neuropsychiatric conditions. These could include but are not limited to pharmacotherapeutics, neuromodulation, psychotherapies, cognitive training, physical exercise, psychosocial interventions, and interoceptive technologies (England et al., 2015; Kim et al., 2018; Swenson et al., 2020; Hertenstein et al., 2021; Schoeller et al., 2024). This would open a venue for developing an optimized patient-tailored approach.

## Author contributions

SA: Conceptualization, Formal analysis, Methodology, Writing – original draft, Writing – review & editing. MB: Conceptualization, Formal analysis, Methodology, Writing – original draft, Writing – review & editing. MC: Conceptualization, Formal analysis, Methodology, Supervision, Writing – original draft, Writing – review & editing.
